# Importance of Indigenous elders’ contributions to individual and community wellness: results from a scoping review on social participation and intergenerational solidarity

**DOI:** 10.17269/s41997-019-00292-3

**Published:** 2020-02-27

**Authors:** Chantal Viscogliosi, Hugo Asselin, Suzy Basile, Kimberly Borwick, Yves Couturier, Marie-Josée Drolet, Dominique Gagnon, Natasa Obradovic, Jill Torrie, Diana Zhou, Mélanie Levasseur

**Affiliations:** 1 School of Rehabilitation, Faculty of Medicine, Université de Sherbrooke, Sherbrooke, Canada; 2 Research Centre on Aging, Centre intégré universitaire de santé et de services sociaux de l’Estrie-Centre hospitalier universitaire de Sherbrooke, Sherbrooke, Canada; 3 School of Indigenous Studies, Université du Québec en Abitibi-Témiscamingue, Rouyn-Noranda, Canada; 4 School of Social Work, Université de Sherbrooke, Sherbrooke, Canada; 5 Occupational Therapy Department, Université du Québec à Trois-Rivières, Trois-Rivières, Canada; 6 Unité d’enseignement et de recherche en sciences du développement humain et social, Université du Québec en Abitibi-Témiscamingue, Rouyn-Noranda, Canada; 7 Department of Public Health, Cree Board of Health and Social Services of James Bay, Montréal, Canada

**Keywords:** Aboriginal people, Indigenous, Elders, Social engagement, Well-being, Health promotion, Autochtone, Aînés, Premières nations, Engagement social, Bien-être, Promotion de la santé

## Abstract

**Objective:**

Wellness is a challenge for Indigenous peoples, partly because Western services do not adopt a holistic approach. By devaluing traditional knowledge, Indigenous values and beliefs, these services lower Indigenous power and affect cultural identities. Indigenous elders participate in intergenerational solidarity by transmitting knowledge, values, and culture in a holistic approach. Despite widespread acceptance of the importance of Indigenous elders’ contributions to wellness, a rigorous synthesis of knowledge has never been done. This study aimed to provide a comprehensive understanding of how Indigenous elders’ social participation contributes to individual and community wellness.

**Method:**

A scoping review was conducted with Indigenous elders and stakeholders in Québec (Canada). Sixteen databases were searched with 57 keywords. Data from the documents retrieved were analyzed, organized, and synthesized based on the International Classification of Functioning, Disability and Health.

**Synthesis:**

A total of 144 documents were examined, comprising 74 scientific papers and 70 sources from the gray literature. Indigenous elders contributed to wellness mainly through relationships and interactions with other community members and non-Indigenous people (72.2%); intergenerational oral and written communications (70.1%); community, social and civic life (45.8%); volunteering and jobs (35.4%); and family life (29.9%). Elders transmit traditional knowledge, strengthen social cohesion, and help to develop positive attitudes such as reciprocity. Their actions favour disease prevention and health promotion, as including traditional approaches increases the acceptability of health and social services.

**Conclusion:**

This scoping review highlights the need for longitudinal studies with mixed-method designs involving Indigenous communities at all stages of the research to deepen understanding of the contributions of Indigenous elders to individual and community wellness.

## Résumé

**Objectives:**

Le mieux-être est un défi pour les peuples autochtones, en partie parce que les services occidentaux n’adoptent pas une approche holistique. En dévaluant les connaissances traditionnelles, les valeurs et les croyances autochtones, ces services réduisent le pouvoir autochtone et affectent l’identité culturelle. Les aînés autochtones participent à la solidarité intergénérationnelle en transmettant les connaissances, les valeurs et la culture dans une approche holistique. Malgré l’acceptation généralisée de l’importance des contributions des aînés autochtones, une synthèse rigoureuse des connaissances n’a jamais été faite. L’objectif de cette étude était de fournir une description approfondie de la façon dont la participation sociale des aînés autochtones contribue au mieux-être des individus et de la communauté.

**Méthodes:**

Un examen de portée a été effectué avec la contribution d’intervenants et d’aînés autochtones de huit des onze nations autochtones du Québec (Canada). Seize bases de données ont été recherchées avec 57 mots-clés. Les données ont été analysées avec la Classification internationale du fonctionnement, du handicap et de la santé.

**Synthèse:**

Au total, 144 documents ont été examinés (74 articles scientifiques et 70 sources de la littérature grise). Les aînés autochtones contribuent au mieux-être principalement par les relations et les interactions avec les autres membres de la communauté et les non-autochtones (72,2 %), les communications intergénérationnelles orales et écrites (70,1 %), la vie communautaire, sociale et civique (45,8 %), le bénévolat et l’emploi (35,4 %) et la vie familiale (29,9 %). Les aînés transmettent les connaissances traditionnelles, renforcent la cohésion sociale et aident à développer des attitudes positives telles que la réciprocité. Leurs actions favorisent la prévention des maladies et la promotion de la santé, car l’inclusion d’approches traditionnelles accroît l’acceptabilité des services de santé et des services sociaux.

**Conclusion:**

Cet examen de portée souligne la nécessité d’études longitudinales avec des devis mixtes impliquant la participation des communautés autochtones à toute étape de la recherche afin d’approfondir la compréhension des contributions des aînés autochtones au mieux-être individuel et collectif.

## Introduction

**Fig. 1:**
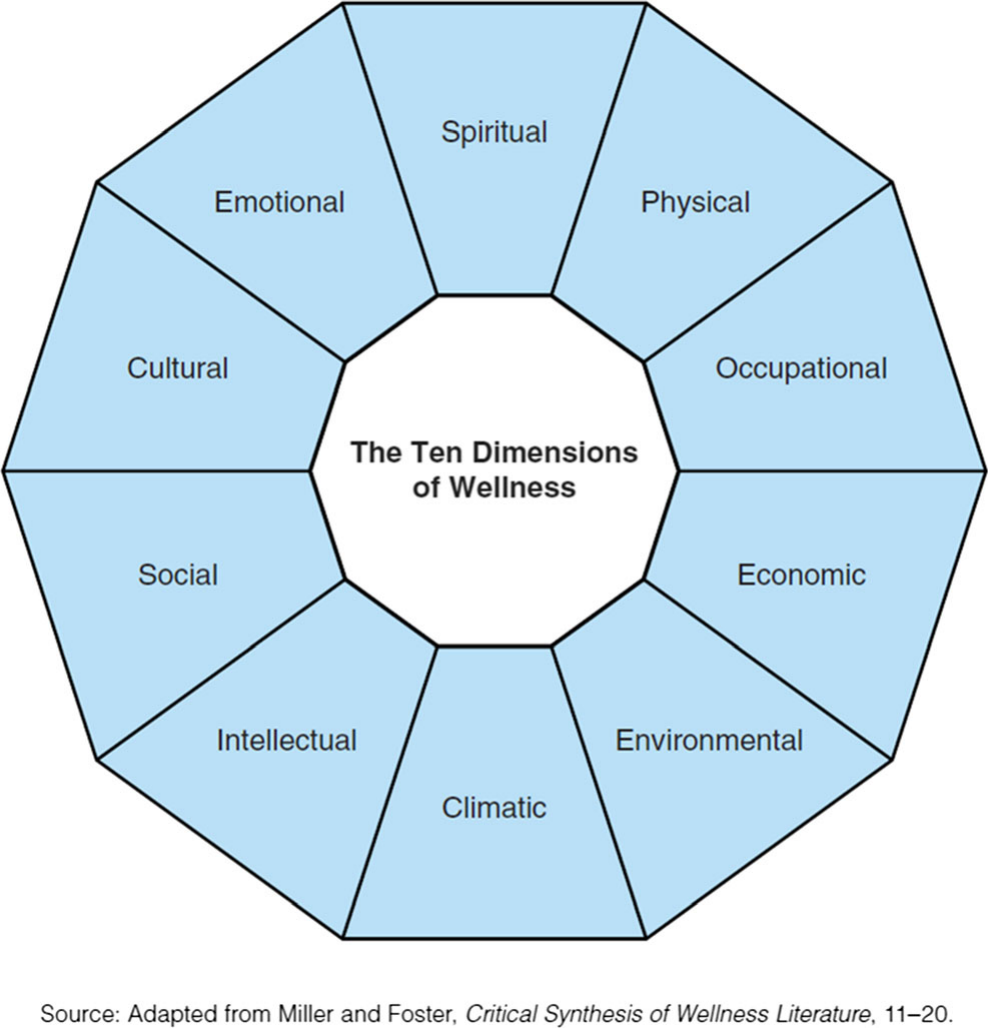
Dimensions of wellness (adapted from Miller and Foster: “Critical synthesis of wellness literature,” 11–20)

Access to proper health and social services is a key issue for Indigenous^1^ peoples, who face significant challenges in education, housing, economic development, well-being, and health (Assembly of First Nations of Quebec and Labrador [AFNQL] and First Nations of Québec and Labrador Health and Social Services Commission [FNQLHSSC] 2007; National Association of Friendship Centres [NAFC] 2013; Saini and Quinn 2013; Wilson et al. 2011). Health and social services delivered using a Western approach have limited success in responding to the needs of Indigenous people, in part because of the complex interactions between the various dimensions of wellness (Saini and Quinn 2013). Moreover, the theories, models, and tools used in health services cause inequities, cultural insecurity, and even harm to Indigenous communities (Drolet and Goulet 2018). Holistic approaches that consider all dimensions of wellness and focus on the strengths of individuals and communities may produce better results than interventions focusing on specific problems that do not consider interactions between dimensions (Institut national d’excellence en santé et en services sociaux [INESSS] 2014). To better meet the needs of Indigenous peoples, promoting the teaching of traditional knowledge and practices in accordance with the United Nations Declaration on the Rights of Indigenous Peoples adopted in 2007 (United Nations 2008) could be part of a holistic approach and contribute to equity in health (Truth and Reconciliation Commission of Canada [TRCC] 2015). Teaching of traditional knowledge and practices involves sharing values, culture, and collective identity, notably through Indigenous elders’ participation in education, community development, and intergenerational relationships (Basile et al. 2017; Kant et al. 2014). In Indigenous contexts, someone is recognized as an elder by other community members based not necessarily on age, but on wisdom, skills, and knowledge (Wilson 2003). Therefore, not all older adults are considered elders and not all elders are older adults. Because elders play an important role in their communities, their social participation and their role in intergenerational solidarity must be considered to develop a holistic approach to individual and community wellness (Miller and Foster 2010). Individual wellness can be defined as a “way of life oriented towards optimal health and well-being in which mind, body, and spirit are integrated by the individual to live life more fully within the human and natural community” (Myers et al. 2000; p. 252). Community wellness is “the simultaneous satisfaction of personal, relational, and collective needs of individuals and communities” (Totikidis 2003; p. 10). The roles implied in favouring individual and community wellness encompass the domains of social participation in the International Classification of Functioning, Disability and Health (ICF) model (World Health Organization [WHO] 2001). Based on a holistic philosophy, the Indigenous worldview includes unity, wholeness, continuation, perpetuity, inseparability, completeness, balance, security, equality, comfort, and health (Graveline 1998) as the many interconnected dimensions of wellness (Fig. 1).

Elders’ social participation and contribution to intergenerational relationships benefit not only themselves but also youth, families, and society (Raymond et al. 2008). Defined as the involvement of a person in activities providing interaction with others in society or in the community, social participation is an important determinant of health and active aging (World Health Organization [WHO] 2001). The ICF describes domains of participation in which people interact with their environment (World Health Organization [WHO] 2001). Scientific evidence has shown that having significant interactions with others is associated with decreased risk for all-cause mortality as well as a range of morbidities. Involving accomplishment in life domains such as interpersonal interactions and relationships, communications, learning, knowledge application and community, and social and civic life (World Health Organization [WHO] 2001), social participation also includes intergenerational solidarities. Intergenerational solidarity refers to mutual help between generations, each one giving to and receiving from the others (Sévigny and Lepage 2016). These solidarities comprise social cohesion based on warmth, affection, attraction, and interaction between generations at the macrosocial and microsocial levels. By its contribution to intergenerational solidarity, Indigenous elders’ social participation can help meet individual and community health and social service needs, address issues, and develop wellness (Kahn et al. 2016).

Previous studies have shown benefits to all dimensions of wellness from Indigenous elders’ social participation in education and health, as well as from intergenerational solidarity in non-Indigenous settings (Olazabal and Pinazo 2010). For youth, families, communities, and elders themselves, benefits encompass not only emotional and spiritual dimensions but also environmental, economic, and cultural dimensions (Kant et al. 2014). Some studies have described Indigenous elders’ involvement in the community (Cross et al. 2010) or their contributions to maintaining Indigenous languages in schools (Kant et al. 2014). Other studies have examined elders’ involvement in the health care system, especially in prevention programs or in the research process to ensure objectives are relevant (Ayunerak et al. 2014). Benefits from elders’ social participation include enhancing education (Souers 1992) as well as promoting health (World Health Organization [WHO] 2001), positive attitudes (Kant et al. 2014), and culture (Wexler 2014). Despite the results of these studies and widespread acceptance of the importance of Indigenous elders’ contribution to wellness, a rigorous synthesis of knowledge has not yet been done. This study thus aimed to provide a comprehensive understanding of how Indigenous elders’ social participation and intergenerational solidarity contribute to individual and community wellness.

## Methods

The six-step method designed by Arksey and O’Malley (2005) was used, combined with an approach centered around principles of research with Indigenous people (Viscogliosi et al. 2017a; Assembly of First Nations of Quebec and Labrador [AFNQL] 2014). The scoping review was conducted in partnership with Indigenous communities and an advisory committee composed of knowledge users (Indigenous organizations and organizations working with Indigenous people, community and public sector partners in health and social services, research partners, and decision-makers).

Two specific questions were addressed:

1. Which domains of Indigenous elders’ social participation have been shown to be associated with or influence wellness at the individual or community level?
2. Which individual and collective benefits are associated with the wellness fostered by Indigenous elders’ social participation?

### Identifying relevant documents

The scientific and gray literature in various databases was searched using keywords (Table 1) validated by the advisory committee (Viscogliosi et al. 2017a). Government and organization reports, audio interviews, videos, video games, and books produced by Indigenous communities, Indigenous organizations, or organizations working with Indigenous people were searched. The research team and advisory committee provided additional documents that did not emerge from the database search.

**Table 1:** Document search strategy

Concepts	Keywords (and French equivalences)	Databases and search engines
Concept A1 Indigenous	Native* OR Indigenous OR “First Nation*” OR Metis OR Inuk OR Inuit OR Eskimo* OR “American Indian*” OR Aboriginal* OR Amerindian*	Autochtonia, First Nations Periodical Index, Bibliography of Native North Americans, Canadian Research Index, Cochrane Database of Systematic Reviews, Medline, CINAHL, Ageline, Sociology database, PsycINFO, Scopus, Academic Search Complete, *Repère*§*, Santecom*§*,* Proquest Research Library, Google / Google Scholar
Concept A2 Elders	Elder* OR Aged [MESH] OR Senior* OR “Old*Adult*” OR “Old age” OR “Old* person*” OR “Old* people” OR “Wise one*” OR Grandmother* OR Grandfather* OR Grandparent* OR “Traditional healer*” OR Leader*	
Concept B Wellness	Resilient OR Resilienc* OR “Capacity building” OR Strength* OR Wellbeing OR “Well-being” OR Wellness OR “Self efficacy” OR “Self esteem” OR “Living conditions” OR Health OR Hardiness [MESH] OR “Indigenous health” [MESH] OR “Psychological wellbeing” [MESH] OR Happiness OR “Self concept” [MESH] OR “Sense of coherence” OR “Socio economic factors” [MESH] OR “Social condition*” OR “World health” [MESH] OR “Global health” OR “Health education” OR “Health promotion”	
Concept C Social participation	Intergeneration* OR Generation* OR “Social participation” OR “Community participation” OR “Social involvement” OR “Social engagement” OR “Community involvement” OR “Community engagement” OR “Civic participation” OR “Consumer participation” [MESH] OR “Community-based participatory research”	

*MESH or *Medical Subject Headings* provides hierarchically organized terminology for indexing and cataloguing biomedical information in databases

The advisory committee and collaborators from Indigenous communities and organizations helped identify gray literature documents, including from Indigenous sources. To do so, invitations were sent to 11 communities from eight different Indigenous peoples (Abenakis, Anishnabeg, Atikamekw Nehirowisiw, Cree, Innu, Inuit, Huron-Wendat and Mohawk) in Québec, Canada, to ask them to participate in the project, collaborate in recruiting elders and representatives, and organize coffee meetings in the communities.

To identify elders’ actions that contribute to individual and community wellness but were not retrieved from the database search, six coffee meetings were held with a total of 65 elders, and individual interviews were conducted with a further 17 elders. Participating elders were between 54 and 84 years old and they had various roles in their communities (Appendix). Preliminary results were presented during these meetings and interviews, which lasted respectively 1.5–5 h and 1–2.5 h. Participants were then asked to suggest additional documents, including reports or newspaper articles reporting elders’ contributions.

### Document selection and data analysis

**Fig. 2:**
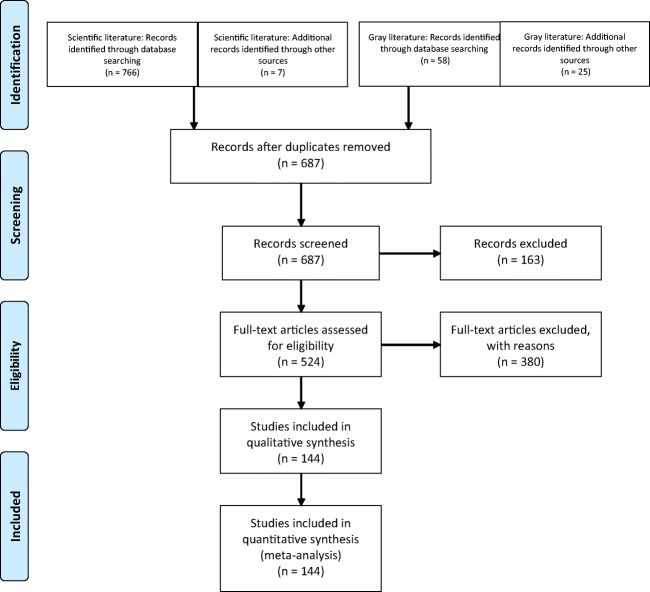
Prisma flow chart for document selection

Two research assistants trained and supervised by the principal investigator and the information scientist separately screened the retrieved documents by title, abstract, and, if in doubt, full text. Following PRISMA guidelines (Moher et al. 2009), and to ensure transparency and the reproducibility of the process, all documents that comprehensively inform the social participation and intergenerational solidarity of Indigenous elders in relation to individual and community wellness were included (Fig. 2). Discrepancies were discussed by the two research assistants and principal investigator to reach a consensus. The remaining ambiguities were discussed with co-investigators. To be retained for analysis, scientific papers had to describe the social participation of Indigenous elders in terms of their involvement or concrete contributions. For the gray literature, documents were retained if they reported benefits of Indigenous elders’ participation, whether through observations or assumptions. Although international scientific papers were included, the gray literature search was limited to the Canadian context for feasibility considerations. The selected documents are available on request.

**Fig. 3:**
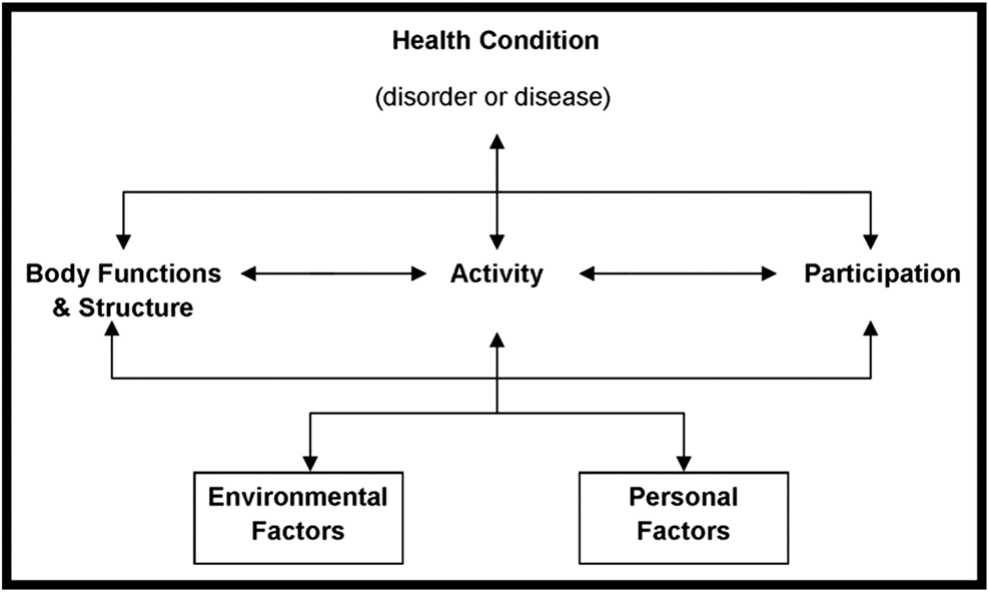
Interactions between components in the ICF (World Health Organization [WHO] 2001:18)

To foster distancing and content validity, the assistants and researchers participating in the analysis shared their preconceptions in relation to the research question before data extraction began. Using a grid based on ICF categories (World Health Organization [WHO] 2001) (Fig. 3), documents were coded by two independent coders for content on social participation, intergenerational solidarity, and wellness. To ensure cultural relevance, the grid was previously discussed and reviewed with members of the advisory committee to recognize, for example, traditional activities as work or volunteering rather than hobbies. A coding guide was used to ensure consistency across analysts. One third of the documents were co-coded by the principal investigator or a co-investigator, and the agreement between coders was 89.5%. The remaining 10.5% coding ambiguities were discussed by the principal investigator and content experts (social participation and Indigenous studies) until 100% consensus was reached. Descriptive statistics on articles and domains of participation were used to define domains of elder participation and benefits, and to explore the difference in coverage between the scientific and gray literature.

Finally, results concerning the contribution of Indigenous elders and their benefits are available in an easy-to-understand form in a written and audio toolkit available in both French and English (Viscogliosi et al. 2017b) that was shared with all Québec Indigenous communities, Native Friendship Centres, First Nations of Quebec and Labrador Health and Social Services Commission (FNQLHSSC), Integrated Health and Social Services Centres (CISSS), Integrated University Health and Social Services Centres (CIUSSS), Indigenous schools, policy-makers, and partner organizations.

## Results

**Fig. 4:**
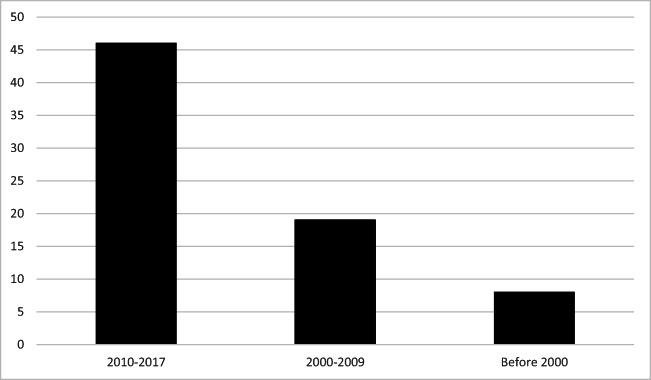
Years of publication for scientific papers

After removing duplicates from the initial 856 documents retrieved, 687 documents were screened by looking at the title and abstract or full text when necessary. Ultimately, 144 were retained, comprising 74 scientific papers and 70 documents from the gray literature (Fig. 4) published between 1962 and August 2017 (the majority (*n* = 46; 62.2%) between 2010 and 2017). Most scientific papers used a qualitative method (*n* = 60; 81.1%). One of the other studies (1.4%) used a pre-experimental design, and six (8.1%) were literature reviews. Of the scientific papers, 27 (36.5%) reported studies in Canada, 35 (47.3%) in the United States, five (6.8%) in Australia, four (5.4%) in Asia, two (2.7%) in Africa, and one (1.4%) in South America. Almost half of the scientific papers focused on people living in Indigenous communities (*n* = 34; 45.9%) while approximately one sixth were based on people living outside communities (*n* = 13; 17.6%), 11 (14.9%) involved people living both inside and outside communities, and the remaining 16 (21.6%) did not specify the location of the population. Gender was not specified in most scientific papers (*n* = 30; 40.5%) or men’s and women’s contributions were not considered separately (*n* = 31; 41.9%). Eight (10.8%) articles included only elder women and one (1.4%) only elder men. No study mentioned lesbian, gay, bisexual, transgender, queer, or two-spirit populations.

Most scientific papers used a convenience sample (*n* = 45; 60.8%) and nearly one quarter did not report sampling type (*n* = 17; 23.0%). Only one (0.7%) document showed negative effects from the contribution of Indigenous elders. Three quarters of the gray literature sources (*n* = 52; 74.3%) did not specify whether the benefits of Indigenous elders’ contribution were assessed or whether they were potential benefits. Finally, social participation was more often detailed in the gray literature than in scientific papers.

### Indigenous elders’ contributions to wellness

The analyses presented in the scientific papers and gray literature, including quantitative and qualitative outcomes and narrative descriptions, show that Indigenous elders contribute to wellness through oral and written communications (*n* = 104; 72.2%); interactions with other community members (*n* = 101; 70.1%); participation in community, social, and civic life (*n* = 66; 45.8%); volunteering and work (*n* = 51; 35.4%); and family life (*n* = 43; 29.9%; Table 2). More specifically, in the *communication* domain, elders promote knowledge transmission, act as consultants, give their opinions and advice about life attitudes and behaviours to adopt towards others, and on practical, moral, and spiritual issues. Through their *relationships and interactions* with family members, youth in schools, and community members in organizations and committees, elders explain Indigenous contexts. In *community, social and civic life*, elders organize and participate in community events such as cultural days and contribute to the inclusion of cultural elements in video games. In addition, elders are involved in *paid work or volunteering*, for example to share their expertise, teach Indigenous languages, participate in the development of employment programs, and integrate traditional healing practices into health centres. In *family life*, elders use medicinal plants, care for grandchildren, and are involved in the resolution of family conflicts. Most elders’ contributions to wellness are aimed at benefiting youth, family, and communities, hence favouring intergenerational solidarity.

**Table 2:** Specific contributions of Indigenous elders to community wellness

**Specific engagement and actions of Indigenous elders (scientific papers) (gray literature)**	
**Communication (55) (49)**	
*Transmission (children, youth, families, communities):*	
◦ Pass on traditional knowledge or stories (52) (42)	
◦ Meeting with youth in school settings (11) (10)	
◦ Stories and legends (5) (7)	
◦ Knowledge (link to the land, identification/usefulness of plants in forests, traditional know-how) (4) (5)	
◦ Encourage spoken language use^3^ (3) (8)	
◦ Life stories (e.g., residential schools, cultural oppression) (2) (2)	
◦ Explanation of journeys for hunting and visiting neighbouring communities (2) (0)	
◦ Audio or video recordings reflecting on culture^3^ (1) (8)	
◦ Proper behaviours towards others (respect, discipline, kindness, reciprocity) (1) (3)	
◦ Translation of plant names, meaning of Indigenous words (1) (1)	
◦ Problem-solving skills (1) (1)	
◦ Know-how to assist childbirth (1) (0)	
◦ Use of humour and metaphors (1) (0)	
◦ Bereavement process^1^ (0) (1)	
*Consultation and opinion*	
◦ Helping to combine traditional beliefs with lifestyle and values (3) (4)	
◦ Listening without judgement (2) (0)	
◦ Accommodation of workplace requirements with traditional culture^1^ (0) (2)	
*Advice*	
◦ Through conversations (including talking circle) (12) (12)	
◦ On traditional health^3^ (3) (6)	
◦ To stay safe, protect and adapt to climate change (2) (1)	
◦ For girls after their first menstruation (2) (0)	
◦ On practical, moral or spiritual issues^3^ (1) (10)	
◦ By writing books^3^ (1) (4)	
◦ During pregnancy (e.g., stay active, eat well) (1) (1)	
◦ On the role of women and family in community health (1) (1)	
◦ To young homeless women (1) (0)	
◦ Through testimony^3^ (0) (8)	
◦ On the meaning of relationship to land, water and the environment^1^ (0) (3)	
◦ By writing songs^1^ (0) (2)	
◦ For Indigenous employees during crises^1^ (0) (1)	
◦ For correctional services (employees, management)^1^ (0) (1)	
◦ By participating in the development of research projects^1^ (0) (1)	
◦ By emceeing public events for non-natives^2^	
◦ Through broadcasting on community radio^2^	
**Relationships and interactions (53) (48)**	
• Relationships with extended family (25) (16)	
• Informal social relationships with community members, including knowledge transmission^3^ (24) (31)	
• Relationships with their children (24) (13)	
• Helping others to take care of themselves (20) (9)	
• Helping others to take care of their health (14) (4)	
• Formal relationships with community members (10) (6)	
• Problem-solving^3^ (3) (8)	
• Decision-making^3^ (2) (10)	
• Informal relationships with people in the same home^1^ (0) (1)	
• Other relationships and interactions^3^ (16) (20):	
• With non-native people: Indigenous culture (oral tradition, traditional dishes, medicinal plants, Indigenous stories, etc.)^1^ (0) (1)	
• Within and outside the nation: sharing wealth (1) (0)	
• With executives and departmental employees: context of Indigenous peoples^1^ (0) (1)	
**Community, social, and civic life (33) (33)**	
• Religion and spirituality (22) (22)	
• Actions to help future generations^3^ (16) (20)	
• Ceremonies (14) (8)	
• Arts and culture^3^ (6) (9)	
• Association^3^ (4) (13)	
• Practice and spiritual teaching, sauna rituals (3) (4)	
• Leisure, games and hobbies (3) (3)	
• Sports (3) (1)	
• Politics^3^ (2) (6)	
• Human rights (2) (1)	
• Organization and participation in community events (e.g., celebrations, cultural days)^3^ (1) (4)	
• Participation in commissions, forums and conferences (1) (4)	
• Contribution to the inclusion of cultural elements in video games (1) (0)	
• Tending of stands (e.g., traditional meals, arts and crafts^3^) at high schools during special events for youth^2^	
• Organization and participation in human chains against Hydro-Québec dams^2^	
• Creation of foundations to help fund sports equipment for youth^2^	
• Organization of “street suppers” to promote communication between elders and youth^2^	
• Planning and participating in weekends in the bush with youth^2^	
• Establishing a clothing counter^2^	
• Animation of a three-month program for survival in the bush (mainly for dropouts)^2^	
• Organization of intergenerational potlucks^2^	
• Animation of baby-naming ceremonies^2^	
• Animation of traditional purification ceremonies at funerals^2^	
**Family life (30) (13)**	
• Custody of grandchildren (7) (6)	
• Use of medicinal plants (4) (6)	
• Walk in the forest to gather plants (2) (3)	
• Traditional family roles (1) (0)	
• Natural adoption^2^	
• Involvement in family conflicts to protect the children^2^	
**Main life domains (work and volunteering) (20) (31)**	
• Traditional and subsistence activities (fishing, trapping, hunting, snowshoeing, gathering plants and berries, gardening, making clothes, crafts)^3^ (15) (28)	
• Healers (4) (3)	
• Tutoring with youth (3) (1)	
• Participation in the development of youth support and employment programs (2) (3)	
• Formal or informal language instruction (2) (2)	
• Education (2) (1)	
• Visits to prisons (1) (3)	
• Acting as a guide (e.g., in museum) (1) (2)	
• Integration of traditional healing practices in health centres (1) (1)	
• Creation of teaching materials for schools (1) (0)	
• Expertise for the Ministry of Education^1^ (0) (1)	
• Filmmakers, writers, playwrights^1^ (0) (1)	
• Homework assistance for elementary school children provided by specialized education college interns and senior high school students^2^	
• Organization of extracurricular activities (e.g., theatre programs for elementary school children)^2^	
• Involvement as project leaders in searching for government grants to fund projects to preserve culture and promote wellness (e.g., conception of a family tree with high school students)^2^	
• Participation in roundtables, committees and gatherings: forestry, culture, health	
• Public safety, education roundtables^2^	
• Expert for the repatriation of traditional objects^2^	
• Member of a Native Friendship Centre^2^	

^1^Only mentioned in the gray literature without being the subject of scientific papers

^2^Only mentioned in coffee meetings

^3^More frequent in the gray literature than in scientific papers

### Benefits from Indigenous elders’ contribution to wellness

The social participation of Indigenous elders was found to benefit youth, families, communities, and the elders themselves. These benefits are seen in support and relationships (*n* = 105; 72.9%), education (*n* = 102; 70.8%), attitudes (*n* = 82; 56.9%), health (*n* = 69; 47.9%), and *product development* (including traditional food, short films, recording and transcription of traditional songs to name a few) (*n* = 33; 22.9%) at individual and community levels (Table 3). More specifically, elders’ contributions benefit youth, family, and communities’ perceived *support and relationships,* such as maintenance and strengthening of family bonds and development of reciprocity and cohesion. The benefits of informal and formal *education* are related to the maintenance of traditional activities; the survival of values and life philosophies such as respect, honesty, and sharing; and the preservation of Indigenous languages and territories. Elders also positively influence individual, relational, and collective *attitudes and behaviours* such as resilience, coping, anger management, hope, perseverance, dignity, motivation, strength, sense of belonging, tolerance, empathy, respect, and pride. *Health* benefits are seen mainly in the prevention of suicide and alcohol and substance abuse and in the promotion of mental health, healthy pregnancy, exercise, and dental health. The promotion of the acceptability of health and social services and the inclusion of traditional approaches to health care and social services are also reported. The *development of teaching, cultural and spiritual products* supporting identity is seen as providing individual and collective benefits for youth, families, communities, and the elders themselves. For example, products such as digital versatile discs (DVDs) foster conservation of traditional knowledge. Finally, the benefits of elders’ contributions are also seen in *services, systems, and policies,* such as in social services (*n* = 21; 14.6%); associative and mutual aid services (*n* = 19; 13.2%); legal services (*n* = 8; 5.6%); architecture, planning, and heritage (*n* = 8; 5.6%); first-necessity services (*n* = 7; 4.9%); communication services (*n* = 4; 2.8%); civic life services (*n* = 3; 2.1%); and civil protection services (*n* = 3; 2.1%). Other benefits are seen in media (*n* = 2; 1.4%), defence of rights (*n* = 3; 2.1%), land claims (*n* = 2; 1.4%), and employment services (*n* = 1; 0.7%).

**Table 3:** Specific benefits of Indigenous elders’ contributions to individual and collective wellness

Specific benefits (scientific papers) (gray literature)	
**Support and relationships (56) (49)**	
• Providing support to extended family (30) (20)	
• Community relations and support^3^ (27) (32)	
• Providing support to close family (24) (12)	
• Maintaining and strengthening family bonds and traditional family structure (19) (3)	
• Relationships with health professionals^3^ (4) (7)	
• Reciprocity (4) (0)	
• Connectivity and cohesion (3) (0)	
• Following advice (2) (2)	
**Services, systems, and policies (51) (42)**	
• Education policies^3^ (50) (52)	
• Health policies (47) (22)	
• Social services policies^3^ (8) (13)	
• Associative and mutual services^3^ (8) (11)	
• Babysitting grandchildren (7) (6)	
• Community development (6) (6)	
• Basic needs policies (5) (2)	
• Legal policies (4) (4)	
• Architecture, planning and heritage material^3^ (2) (6)	
• Human rights (2) (1)	
• Communication policies^3^ (1) (3)	
• Citizen participation policies^3^ (1) (2)	
• Media policies (1) (1)	
• Participation in juries (1) (0)	
• Establishment of a television network^1^ (1) (0)	
• Employment policies (1) (0)	
• Planning and developing open-air recreational areas, protected areas and rural areas^1^ (1) (0)	
• Social security policies and civil protection^1^ (0) (4)	
• Social harmony^1^ (0) (3)	
• Civil protection policies^3^ (0) (3)	
• Land claims^1^ (0) (2)	
• Preparation of conditional release hearing^1^ (0) (1)	
• Employment reintegration program for prisoners^1^ (0) (1)	
**Formal and informal education (50) (52)**	
• Cultural knowledge, traditional values (e.g., honesty), regulations, and life philosophy^3^ (36) (41)	
• Role modeling (22) (11)	
• Identity (18) (17)	
• Indigenous language and territory (14) (15)	
• Activities and traditional practices (cooking, sewing, knitting, fishing, picking, hunting, dancing) (10) (11)	
• Learning by seeing, learning by doing, learning by sharing, intellectual knowledge transfer (7) (9)	
• Concepts, values, perceptions, behaviours, verbal and non-verbal communication, interpersonal relations (5) (7)	
• Encouraging perseverance to higher education levels (2) (0)	
• Inspiring educational programming suitable for reality (1) (2)	
• Fauna and flora (1) (1)	
• Philosophical approach of education programs^1^ (0) (2)	
**Health (47) (22)**	
• Traditional medicine. Healing with medicinal plants (14) (11)	
• Traditional treatment of hemorrhage in childbirth^1^ (0) (1)	
• Promotion of health and well-being (14) (6)	
◦ Culturally healthy life (14) (6)	
◦ Dental health (2) (0)	
◦ Exercise (i.e., dance) (1) (0)	
◦ Mental health^1^ (0) (1)	
◦ Advice for a healthy pregnancy^1^ (0) (1)	
◦ Immunity against diseases^1^ (0) (1)	
• Prevention (14) (5)	
◦ Substance abuse (alcohol and drugs) (11) (4)	
◦ Junk food (5) (2)	
◦ Suicide (4) (1)	
◦ Smoking tobacco (2) (0)	
◦ Sexual abuse^1^ (2) (0)	
◦ Gambling (1) (0)	
◦ HIV/AIDS (1) (0)	
◦ Delinquency (0) (1)	
◦ Behaviours related to lifestyle^1^ (0) (1)	
• Newborn care (4) (1)	
• Health professionals’ behaviours^3^ (2) (3)	
• Childbirth support^1^ (2) (1)	
• Help in accepting interventions and learning to trust health services (1) (0)	
• Multi-sectoral collaboration with teachers, researchers, police, youth and community members^1^ (0) (1)	
• Therapy co-animation^1^ (0) (1)	
**Attitudes and behaviours (44) (38)**	
• Personal	
◦ Strength development (spiritual, cultural, family-related)/life vision (18) (10)	
◦ Resilience (14) (4)	
◦ Wisdom, balance, harmony (8) (4)	
◦ Healing (philosophical, psychological, spiritual, social)^3^ (7) (10)	
◦ Independence and perseverance (4) (6)	
◦ Self-confidence, self-esteem (4) (5)	
◦ Consciousness/transformation/integration (3) (0)	
◦ Grieving (2) (2)	
◦ Forgiveness (1) (2)	
◦ Philosophy, optimism (1) (2)	
◦ Discipline (1) (1)	
◦ Catharsis (1) (0)	
◦ Meaning in life (1) (0)	
◦ Adaptability^1^ (0) (1)	
◦ Hope^1^ (0) (2)	
◦ Inner peace^1^ (0) (2)	
◦ Anger management^1^ (0) (1)	
◦ Liberation^1^ (0) (1)	
◦ Motivation, energy and passion^1^ (0) (1)	
◦ Dignity^1^ (0) (1)	
• Relational	
◦ Extended family behaviours (12) (4)	
◦ Close family behaviours (10) (5)	
◦ Respect^1^ (4) (4)	
◦ Sense of belonging (1) (0)	
◦ Responsibility (personal, family/community) (0) (4)	
◦ Empathy^1^ (0) (2)	
◦ Kindness^1^ (0) (1)	
◦ Preservation of peace between families^1^ (0) (1)	
◦ Tolerance^1^ (0) (1)	
◦ Patience^1^ (0) (1)	
◦ Youth’s sense of purpose towards elders^1^ (0) (1)	
• Collective	
◦ Community behaviours^3^ (9) (16)	
◦ Societal norms (9) (11)	
◦ Societal behaviours (6) (3)	
◦ Proudness (3) (6)	
◦ Rooting in culture (2) (3)	
◦ Collective memory and conscience (1) (2)	
◦ Economic security (1) (1)	
**Product development (20) (13)**	
• Educational material development (10) (6)	
• Religious/spiritual material development (4) (4)	
• Food product development (4) (4)	
• Cultural and leisure products development^3^ (3) (8)	
• Story writing^3^ (1) (4)	
• Communication products development^3^ (1) (2)	
• Sweat lodge construction^1^ (0) (3)	
• DVD recording for traditional knowledge (i.e., traditional handcrafts)^1^ (0) (2)	
• Recording and transcription of traditional songs^1^ (0) (1)	
• Short film production and participation^1^ (0) (1)	
• Transcription of elders’ conferences explaining traditional medicine and other traditional knowledge^1^ (0) (1)	
◦ Preparation of traditional food^1^ (0) (1)	

^1^Only mentioned in the gray literature

^2^Only mentioned in coffee meetings

^3^More frequent in the gray literature than in scientific papers

The results of this scoping review revealed some differences between contributions found in scientific papers and in the gray literature. The contributions of Indigenous elders in specific domains of social participation such as decision-making, traditional activities, association, craft, and politics received less attention in scientific papers than in the gray literature (Table 2). Similarly, some benefits such as maintaining cultural knowledge, traditional values, rules of life, and truths of life, as well as promoting community attitudes and cultural and leisure product development, appeared less often in scientific papers than in gray literature sources (Table 3). Several values of great importance to Indigenous people, such as passion, dignity, sense of responsibility and emotional balance (11,50,52), were not included in scientific papers. Also, actions in civil protection policies are mentioned only in the gray literature.

## Discussion

### Elders’ social participation, intergenerational solidarity, and contributions to wellness

This study aimed to provide a comprehensive understanding of the characteristics and contributions of Indigenous elders’ social participation to individual and community wellness. Overall, Indigenous elders’ social participation occurs in relationships and interactions; in different forms of communication; in work and volunteering; and in social, community, civic, and family life. Benefits mainly occur through the maintenance of interpersonal, family, and conjugal relationships and the social support received by youth, families, and communities. Other benefits include having access to educational and cultural products made by elders; individual and collective attitudes; physical and mental health; and in the development of services, systems, and policies.

Most Indigenous elders’ contributions were found in relationships and interactions with family and other community members (Wexler 2011). These results are in line with those of a large study involving 16,369 non-Indigenous seniors showing that family or friendship activities outside the household are among the most popular involvements for seniors (Statistics Canada 2015). Frequent elders’ participation through relationships and communication is not surprising because oral tradition is the primary mode of Indigenous knowledge transmission (First Nations and Indigenous Studies 2009). Elders expressed the importance of being able to pass on knowledge to the youth, both at the individual level and within the community (Government of Canada 2015). Elders also appreciate when youth teach them about technology and other modern tools. In addition, strong relationships between elders and youth appear to have a positive impact on community health and wellness (Basile 2017).

Intergenerational trauma related to residential schools affects Indigenous people from all generations, including elders (Bombay et al. 2014). Elders are vital to communities, and their specific needs must be addressed. Indeed, reciprocity is important in traditional teachings, which is the value of giving back to those who are helping fellow community members (Assembly of First Nations of Quebec and Labrador [AFNQL] 2014). To support the contribution of elders to individual and community wellness, it is important to increase access to resources and services such as individual and group interventions which may include speech circles (Justice réparatrice du Québec 2019) or interventions with psychologists, social workers, and occupational therapists favouring the development of elders’ strengths. The participation of elders as co-therapists is often an important facilitator. Other ways of increasing elder participation, such as the Personalized citizen assistance for social participation (APIC; Levasseur et al. 2016), may help increase elders’ roles according to their values and interests.

Some community members seeking guidance from elders are undergoing trauma (National Inquiry into Missing and Murdered Women and Girls 2019). Because community members having been victims of violence may be likely to inadvertently perpetuate those patterns, safety measures must be implemented to prevent traumatizing practices. The Cree Women of Eeyou Istchee Association developed a project on mistreatment prevention. The *Guide de référence pour contrer la maltraitance envers les personnes aînées* (Gouvernement du Québec 2016) includes a section on best practices and a specific approach for First Nations. The Governmental Action Plan to Counter Elder Abuse (Ministère de la famille – Secrétariat aux aînés 2017) recommends several measures to prevent, identify, and diminish mistreatment. The website of the Canadian Network for the Prevention of Elder Abuse contains webinars, publications on best practice tools, community tools, and a blog on new initiatives in mistreatment prevention. In addition, the *IN HANDS – practical guide* supports the actions of professionals who intervene in cases of elder abuse. The “*Aide Abus Aînés*” line answers questions from victims and witnesses of abuse.

### Cultural sensitivity and safety

The National Aboriginal Health Organization suggests that Indigenous and non-Indigenous professionals and educators must communicate according to the person’s social, political, linguistic, and spiritual contexts (Baba 2013). Cultural safety allows persons to perceive that services are respectful of their values and beliefs as well as of their cultural identity (Gerlach 2012). Cultural sensitivity is the recognition of the necessity to respect cultural differences (Baba 2013). As Indigenous elders transmit knowledge, they could play a key role in training the professional and managerial staff working with individuals and groups to promote culturally sensitive approaches (Nametau Innu 2010). Enhancing cultural identity and healing power is important to wellness (Government of Canada 2013). Identity is a key factor in child development, as a sense of belonging to family, community, and peers helps children deal with adversity. Identity can also help to decolonize practices by improving understanding of the relationship to the land and spiritual traditions (Iseke 2013). The social participation of elders is a promising way to increase Indigenous involvement in the governance of services, as encouraged by the Truth and Reconciliation Commission of Canada (Truth and Reconciliation Commission of Canada [TRCC] 2015). During coffee meetings, Indigenous elders reported that when they are questioned, only their perceptions of problems are considered, although they could also suggest solutions. This highlights cultural differences: Western-centred cultures focus on problem diagnosis whereas Indigenous cultures focus on reconstruction and resilience. Partnerships must be developed with Indigenous people to help build research projects based on their needs so that they obtain tangible benefits. Researchers, decision-makers, and professionals have to design interventions that meet the specific needs of different groups within Indigenous communities. Varying from one community to another, these interventions must ensure cultural safety oriented towards values, customs, and needs and be offered to all Indigenous people whether they live on the land, in communities, or in urban settings.

### Valuing epistemologies and Indigenous voices in future research

Many of the elders’ contributions to individual and community wellness were mentioned more often in the gray literature than in scientific papers. Moreover, some variables that are meaningful for Indigenous people, particularly relating to the development of positive attitudes to wellness such as passion, dignity, sense of responsibility, and emotional balance, were not mentioned in scientific papers. This could be because the Western discourse has come to prioritize the written word even though oral-based knowledge systems are predominant among Indigenous peoples (Payne et al. 2013). The inclusion of oral knowledge in research constitutes a challenge as it requires trust from the Indigenous person (Indigenous Corporate Training 2018). The Supreme Court of Canada recognized the voice of elders as receivable proof for the first time in 1997 with the Delgamuukw ruling. Ignoring Indigenous epistemologies and imposing a Western vision of knowledge harms Indigenous people. Moreover, generating knowledge in a paternalist and colonial way damages current and future relations with Indigenous communities (CRSH, CRSNG et IRSC 2014). In addition, scientific measurement tools are often not developed in partnership with Indigenous people and thus may not cover all relevant aspects of wellness (Morison 2004). As some of the wellness dimensions in the gray literature were not mentioned in scientific papers, partnership research should measure the most meaningful variables for Indigenous peoples in relation to the different components of wellness. Measurement tools could be developed or validated with Indigenous people to identify important elements of social participation as well as dimensions of wellness (Basile et al. 2018). Because language and identity are major issues for Indigenous peoples (Drolet and Goulet 2018; Itinnuaq and Itinnuaq 2007), these aspects should be considered in research and interventions aimed at wellness. The results of this scoping review are aligned with call to action 22 of the Truth and Reconciliation Commission of Canada (Truth and Reconciliation Commission of Canada [TRCC] 2015) that recognizes Indigenous healing practices in the social services and health system, also with call to action 48 promoting self-determination of Indigenous peoples in the development of interventions towards wellness, and finally with call to action 53 encouraging the development of an action plan for research favouring reconciliation. As Indigenous people value processes as much as results (Payne et al. 2013), future research should make greater use of mixed-method designs that not only measure the effects of elders’ contributions (quantitative component) but also understand their process (qualitative component). In accordance with ethical principles of research with Indigenous people (Asselin and Basile 2012), partnership approaches should value their knowledge, creativity, and strengths to generate tangible benefits (Government of Canada 2015; Assembly of First Nations of Quebec and Labrador [AFNQL] 2014). In such research processes and in the development and delivery of care, services, and interventions, Indigenous elders and representatives should be actively involved in sharing traditional knowledge recognized as best practices by Indigenous people (Nametau Innu 2010; Government of Canada 2013). Meanwhile, services could be developed in a holistic approach to wellness by focusing on the development of strengths rather than targeting issues that ignore the context.

Although the ICF (World Health Organization [WHO] 2001) is a universally accepted model of social participation, cultural validation of the ICF with Indigenous peoples should be considered in future research. In the development of the coding grid based on the ICF model, the classification of certain traditional activities was difficult. Results from studies based on other definitions and models of social participation were included to broaden the understanding. Epistemologies and axiologies at the foundation of Western-centric theories, models, and tools used in health can cause harm to Indigenous people by creating cultural insecurity and even devaluing their power and cultural identity. This scoping review shows ways to improve the ICF to make it more respectful of Indigenous knowledge, values, and beliefs.

Although many scientific papers reported the contribution of Indigenous elders to individual and community wellness, there was little robust evidence of the benefits of elders’ social participation and intergenerational solidarity. In the gray literature, Indigenous elders’ participation reported benefits based on observations or assumptions. To avoid perpetuating epistemic injustices (Fricker 2007), epistemic resources must be part of literature reviews to make sure that Indigenous voices are valued and contribute to knowledge development. Further research must be conducted to analyze the effects of interventions involving the social participation and intergenerational solidarity of Indigenous elders. Such research should preferably be carried out using representative sampling strategies, taking gender into account (Quebec Native Women Association [QNWA] 2012), distinguishing seniors from elders, and using outcome measures that are meaningful for Indigenous peoples. Because most of the studies conducted to date used a cross-sectional approach, future research aimed at monitoring the development of wellness with regard to social participation and intergenerational solidarity should use longitudinal designs.

### Strengths and limitations

The multidisciplinary research team involved stakeholders from public and community sectors to enhance the relevance of the research question, the inclusion of significant sources, and the validity of the analysis. The contribution of key informants (e.g., Indigenous elders and representatives, health centres, band councils, decision-makers) enabled the team to retrieve relevant documents that could not be found using conventional scientific search methods. The inclusion of gray literature valued by Indigenous peoples promotes mutuality in the knowledge transmission process (Government of Canada 2013; Nametau Innu 2010) and favours epistemic justice (Fricker 2007). The systematic scoping review method using co-validation increases the reproducibility of the results. Knowledge translation was supported by principles and approaches that enhance collaboration between researchers and knowledge users (Arksey and O’Malley 2005). Through these results, partnership initiatives with communities, policy-makers, stakeholders, and researchers from different disciplines can lead to innovative and culturally sensitive research proposals.

As in other scoping reviews, the aim was not to assess the quality of the studies and the results thus include data obtained using various methods, each with its own strengths and drawbacks. It is also possible that positive results are overrepresented (publication bias). Even though the scoping was extended to include Indigenous sources, for feasibility reasons, the gray literature search was limited to French and English documents and to the Canadian context, and the search strategy involved a limited number of keywords. Some topics not covered in the retrieved documents could be important in documenting Indigenous elders’ contribution to individual and community wellness. In addition, although this article focuses on elders’ contribution to wellness, further studies should consider the contribution of younger individuals who also hold traditional knowledge and have complementary viewpoints. Finally, different Indigenous languages and cultures are likely associated with a variety of elder actions among communities and peoples who were not addressed in this scoping review.

## Conclusion

This scoping review shows that Indigenous elders contribute to individual and community wellness. Benefits of the social participation of elders were found in support and relationships; education; attitudes; health; development of products; and contribution in systems, services, and policies. Sharing these results with Indigenous communities, organizations, and decision-makers could lead to increased use of holistic approaches that focus on the contribution of Indigenous elders in enhancing individual and community wellness and cultural security in healthcare. It is essential to work with communities to support the implementation of intergenerational actions involving elders in order to overcome existing health and social challenges based on a holistic approach favouring cultural security. There is also a need to promote facilitators and reduce barriers to support the current actualization of elders’ contributions. Results from this scoping review may support best practices and the development of innovative inclusive public health and social services interventions, including clear guidelines on how to promote wellness. The results also highlight the need for more longitudinal mixed-method designs involving Indigenous communities at all stages of the research. This scoping review is the first stage of a wider research program aiming to (1) describe the factors that foster and impede Indigenous elders’ contribution to wellness, (2) design intergenerational programs promoting Indigenous elders’ contribution to wellness, (3) follow the evolution of individual and community wellness with culturally relevant methods and tools to promote cultural safety and capture significant aspects of Indigenous wellness, such as identity and language, and (4) explore ICF validity in Indigenous contexts.

In the current context characterized by the aging of Western populations and the devaluation and even mistreatment of Western elders, this scoping review highlights a wisdom that Western societies would do well to examine for inspiration. Indigenous elders’ wisdom is key to supporting the health and wellness not only of elders themselves but also of individuals and communities, as well as intergenerational solidarity.

## Appendix. Roles of elders who participated in the study

- Cultural coordinator
- Member of the board of directors of a native friendship centre
- Museum director
- Spiritual leader
- Volunteers for homework in primary school
- First Nations language teachers
- Guides for land-based shipments
- Speaker
- Representatives of elders’ committees
- Teachers of traditional activities
- Former politicians

## Footnotes

1 The term Indigenous does not do justice to the inherent and unique diversity of the Indigenous peoples in Canada but is used for the sake of simplicity. In Canada, Indigenous peoples include First Nations, Inuit, and Metis.
